# Boosting Contraceptive Uptake in Urban Uganda: Older Women Benefit When Layering Adolescent and Youth Interventions Onto Existing Family Planning Programming

**DOI:** 10.9745/GHSP-D-22-00308

**Published:** 2024-05-21

**Authors:** Albert Bwire, Denis Joel Sama, Jessica Mirano, Paul Nyachae, Kenneth Owino, Josephine Nabukeera, Juliet Tumuhairwe, Maheen Malik, Ian Salas, Vanessa Mitchell, Krishna Bose

**Affiliations:** a Jhpiego Uganda, Kampala, Uganda.; b William H. Gates Sr. Institute for Population and Reproductive Health, Baltimore, MD, USA.; c Jhpiego Kenya, Nairobi, Kenya.

## Abstract

**Introduction:**

Uganda has a large young population with a high unmet need for family planning (FP). Although there have been many efforts to improve access to and uptake of contraception, improvements have been slow. The Ministry of Health Uganda partnered with The Challenge Initiative (TCI) to implement a novel multipronged approach layering adolescent and youth sexual reproductive health (AYSRH) onto a functioning general FP program for women of reproductive age in 3 local governments of Buikwe, Mukono, and Iganga. We describe the approach and aim to determine whether layering AYSRH interventions onto an existing program resulted in increased contraceptive uptake among adolescents and youth aged 10–24 years and among women aged 25–49 years.

**Methods:**

We analyzed service statistics from the Uganda Health Management Information System to assess contraceptive uptake for adolescents and youth (aged 10–24 years) and older women (aged 25–49 years) before and after the implementation of the AYSRH approach in 3 areas (Buikwe, Iganga, and Mukono) compared to 11 areas where only the general FP program was implemented and the Uganda country total.

**Results:**

This analysis showed that before the start of TCI’s support, levels of contraceptive uptake were similar in all local governments. However, after implementation, there was an increase in uptake for general FP program only areas (1.7-point advantage over country total) and an even greater increase in general FP+AYSRH areas (2.4-point advantage over FP only programming). This was observed in both adolescents and youth aged 10–24 years and among women aged 25–49 years.

**Conclusion:**

The layering of TCI’s AYSRH interventions onto a well-functioning FP platform not only increased contraceptive uptake among adolescents and youth aged 10–24 years but also boosted uptake among women older than age 25 years.

## Abstract (precis)

Layering interventions designed to increase access to contraceptive uptake for adolescents and youth onto existing family planning programming resulted in boosting uptake for older women as well as adolescents and youth.

## Teaser

**Key Messages:**

- To address the high unmet need for contraception for adolescents and youth in Uganda and address the barriers to contraceptive access and uptake, particularly in urban areas and informal settlements, adolescent- and youth-specific focused activities were added to the advocacy, service delivery, and demand generation components of an already functioning general family planning (FP) program.
- After the interventions were implemented, contraceptive uptake increased for adolescents and youth aged 10–24 years as well as older women aged 25–49 years, demonstrating that all women, not just youth, benefit when youth-focused interventions are put in place to improve community norms, decrease provider bias, and increase demand for contraceptives.
- Intentional layering of an adolescent and youth sexual reproductive health (AYSRH) component onto an already functional FP program targeting women of reproductive age allows for rapid implementation of adolescent- and youth-specific interventions through the same systems, which, in turn, spurs uptake.

**Key Implication:**

- General FP programs that include a special focus on AYSRH experience increases in contraceptive uptake for adolescents and youth and a boost in uptake for older women.

## INTRODUCTION

Of the estimated 190 million women of reproductive age (WRA; aged 15–49 years) across the world who do not use modern contraceptives, a majority (83%) are in sub-Saharan Africa.^1^ Contraceptive use in Africa is estimated at 36% compared to at least 65% in the rest of the world,^2^ while unmet need for family planning (FP) in Africa is estimated at 22% compared to 15% in Oceania and below 10% in rest of the world.^2^ Uganda conforms to this pattern, with over 28% of currently married women and 32% of sexually active unmarried women having an unmet need for FP.^3^^,^^4^ Low contraceptive use has far-reaching consequences, including unplanned pregnancies, and indirect impacts, such as avoidable maternal deaths and illnesses, unsafe abortions, and child morbidity and mortality.^2^ For Uganda, country-level estimates indicate that 52% of pregnancies are unwanted or mistimed, with over 43% of unintended pregnancies attributable to the unmet need for FP.^3^^,^^4^ Among women wanting to avoid pregnancy in low- and middle-income countries, unmet need is much higher for adolescents than for all women aged 15–49 (43% vs. 24%).^5^ This is even more problematic for Uganda, with 22% estimated to be youth aged 15–24 years, while in sub-Saharan Africa overall, youth make up 20% of the population.^6^ An estimated 25% of Ugandan women aged 15–19 years have begun childbearing, and 18% of this population reported initiating sex by age 15 years and 62% by age 18 years.^7^^,^^8^ In addition, the use of modern contraception by WRA in the country was 30% in 2018 and 34% in 2021.^9^ But for adolescent females, only 9.4% of those aged 15–19 years and 28.3% of those aged 20–24 years used a modern method of contraception in 2018.^8^

Unmet need is much higher for adolescents in low- and middle-income countries than for all women aged 15–49 years and particularly in Uganda, with 22% estimated to be youth aged 15–24 years.

In this context, increasing the coverage of effective FP and contraceptive services to improve access and use for WRA, particularly adolescents, is essential and has been shown to be affected by several supply-side and demand-related factors. Health system challenges continue to affect the provision of quality FP services,^10^ with frequent stock-outs of FP commodities that are exacerbated by inadequate health provider skills and other service quality factors. On the demand side, contraceptive uptake is negatively affected by inadequate awareness and knowledge of different FP options among WRA and adolescents, community perceptions and biases regarding specific contraceptive methods, inadequate trust in the health system compounded by provider bias, and the cost and limited availability of services.^11^^–^^13^

Although urban areas are often considered and reported to be better off due to the wider availability of and apparent proximity to services,^14^ the increasing intra-urban inequalities may affect access to and use of FP services.^14^^,^^15^ In fact, over 70% of all urban residents in sub-Saharan Africa live in informal settlements, which are characterized by extreme poverty and have poor maternal and child health indicators.^16^ Moreover, urban areas—and particularly informal settlements—often lack urban primary health care structures. Developmental focus has typically been on rural areas, resulting in limited free, quality primary health care in urban areas (though private health care facilities proliferate),^17^ which further affects access to and use of FP services. In general, for adolescents and youth, the factors influencing their use of contraception include parental disapproval, peer influences, the potential negative outcomes of contraceptives compounded by inadequate knowledge, sociocultural barriers, and stigma.^18^^,^^19^

In addition to these challenges facing the urban poor in Uganda, the inadequacy of the primary health care services, although free, means accessing the prolific private “shops” that are not free. But without financial means, the choice that remains is the non-use of contraceptives,^20^ thereby leaving them at an elevated risk of teenage pregnancy and sexually transmitted infections.^21^

To address these barriers, in 2017, The Challenge Initiative (TCI) commenced its support for urban local governments and health systems in Uganda to help improve their capacity to implement high-impact and sustainable interventions for FP.^22^ In 2018, the focus was enhanced on adolescents and youth in selected geographies that were already supporting FP interventions in response to demands from local governments concerned about increasing unintended pregnancies among this cohort of women aged 15–24 years.^23^ The goal was to improve contraceptive access for adolescents and youth aged 15–24 years within the larger cohort of women aged 15–49 years already being supported by its FP program.

A review on improving access to and use of contraception by adolescents^24^ highlights the factors previously discussed that hinder or support these efforts, which TCI addresses in its program support. However, this review did not distinguish between the relative effectiveness of FP programs for older women and AYSRH programs when combined or layered in temporal sequence, as was supported by TCI. In fact, we have found no published literature that documents the effects of layering AYSRH interventions on an existing and successful FP program and how that affects contraceptive uptake for adolescents and youth as well as older women. As noted previously, for sub-Saharan Africa and Uganda, contraceptive use among WRA and adolescents and youth remains low due to multiple intersecting factors. Improving contraceptive uptake and use for WRA is in itself a challenge that TCI continues to address.^22^ Adding a focus on increasing contraceptive rates for adolescents and youth is an even more sensitive issue^18^^–^^20^ that can create community backlash.^25^ Therefore, TCI partnered at governance, facility, and community levels with adolescents and youth^23^ to ensure acceptance and support for AYSRH-focused interventions with gatekeepers and adolescents and youth themselves.

We describe an ecological study that sought to determine whether layering AYSRH interventions onto an existing and functioning FP program results in increased contraceptive uptake among 2 groups: (1) adolescents and youth aged 15–24 years and (2) women aged 25–49 years. Ecological studies are observational studies that relate exposure and outcomes at the population level.^26^

## THE CHALLENGE INITIATIVE PROGRAM DESCRIPTION AND INTERVENTIONS

TCI, funded by the Bill & Melinda Gates Foundation, is a development platform supporting local governments in urban settings to rapidly scale up evidence-based FP and AYSRH interventions for the urban poor.^22^^,^^23^ The TCI model prioritizes sustainability and local ownership and uses a health systems approach for scaling high-impact interventions and practices.

In July 2017, the TCI program began implementation in urban Uganda, initially focusing on WRA (aged 15–49 years) in 14 local governments. This general FP program comprised 3 program areas: advocacy, service delivery, and demand generation (Table 1). In July 2018, to address the barriers to contraceptive uptake previously mentioned, the program began layering AYSRH program interventions focusing on adolescents and youth aged 15–24 years in addition to the general FP program in 3 of the 14 local governments: Buikwe, Iganga, and Mukono.

**TABLE 1.** Comparison of General FP Program Interventions and Additional AYSRH Interventions in Three Ugandan Local Governments

	**General FP Program Only**	**General FP+AYSRH Program**
Advocacy	Increased government allocations to FP	Increased government financial allocations for FP and AYSRH
	FP champions	Youth engagement: • Governance level: monthly program implementation team meetings through youth champions • Community level: intergenerational dialogues with parents, community leaders, and key elder influencers With interfaith religious leaders discussing AYSRH
Service delivery	Whole-site orientations related to FP, including commodity and data management	• Additional whole-site orientations for reducing bias toward youth who access contraceptives and dispelling myths about the appropriateness of all methods
	Health facility strengthening through 72-hour makeovers	Quality improvement through application of national standards for adolescent- and youth-friendly health services and supervisory checklists, with specific attention to reducing health provider biases
	Integrated FP outreaches^a^ and in-reaches^b^	Integrated youth-focused outreaches^a^ and in-reaches^b^
	Fixed-day services at facilities	Additional fixed-day services for youth in the same facilities as fixed-day services for general FP program
	Supportive supervision on providing FP methods	Supportive supervision by district quality assessment teams emphasized the provision of all contraceptive methods including long-acting reversible contraceptive methods; assessment and implementation of the quality checklist
	Postpartum FP for all women	• Postpartum FP for young mothers (particularly unmarried teenagers) Pharmacy engagement and training on reducing bias and eliminating myths and misconceptions about AYSRH and ensuring referrals for long-acting reversible contraceptive methods
Demand generation	Counseling and referrals through trained community health volunteers	• Counseling and referrals through trained community health volunteers, who include youth recruits and youth champions
	Male engagement initiatives	• WhatsApp groups for youth • Social mobilization through community events • Radio segments on AYSRH

Abbreviations: AYSRH, adolescent and youth sexual and reproductive health; FP, family planning.

^a^ An integrated FP outreach is a health service delivery activity done in the community away from the facility to bring contraceptive services closer to the community. The services are provided at the community level within locally available venues—such as schools, social halls, workplaces, community grounds, markets, and religious facilities—focusing on both men and women of reproductive age.^27^

^b^ In-reaches describe the provision of FP services to clients mobilized from the community to a particular facility, usually a primary health center, on designated days of the week. The facility providers where in-reaches occur work closely with the community social mobilizers to synchronize mobilization days to clinic days for seamless service provision.^28^

As noted, youth are not always health literate and often are not aware that their neighboring public health facilities may have improved the quality of service delivery.^15^ TCI partnered with local governments to integrate youth champions, who not only advocated for AYSRH at the regular government program implementation team meetings but also were engaged in informing their peers about AYSRH.^27^ This increased the demand for quality contraceptive services at community outreaches and facility in-reaches in those facilities that had undergone a youth-focused whole-site orientation.^28^^,^^29^ The youth champions used social media and traditional communication approaches and even sometimes functioned as community health volunteers. Several youth champions were young men who were instrumental in reaching other young men who are frequently overlooked in FP programs. Table 2 summarizes the number of activities that TCI supported for FP and additional AYSRH-focused efforts in Buikwe, Iganga, and Mukono, as well as the other 11 FP only local governments.

**TABLE 2.** FP and AYSRH-Focused Activities Conducted in Uganda, December 2018–June 2021

	**Addressing Health Service Delivery**					**Addressing Community Norms**	
	**Integrated FP Outreaches, No.**	**FP In-Reaches, No.**	**AYSRH Outreaches, No.**	**AYSRH In-Reaches, No.**	**Providers Trained on Bias Reduction and Coaching on Improved FP Approaches, No.**	**Intergenerational Community Dialogues, No.**	**Interfaith Community Dialogues, No.**
Buikwe	33	14	20	4	252	24	27
Iganga	17	14	20	7	500	5	7
Mukono	31	20	20	1	28	19	5
General FP local governments	365	111	19	2	1,976	0	0

Abbreviations: AYSRH, adolescent and youth sexual and reproductive health; FP, family planning.

Youth champions were integrated to advocate for the government’s AYSRH program.

## METHODS AND ANALYSIS

To respond to the research question, we used available data from Uganda’s Health Management Information System (HMIS) in this ecological study. Ecological studies are observational studies that relate exposure and outcome at the population level instead of at the individual level. Our article does not rely on cross-sectional data from household or women surveys but instead uses population-level FP service statistics data from HMIS. While it is difficult to remove confounding variables in this type of research, it is valuable for using population data that are already available and understanding relationships among variables of interest.^26^

We collected data on FP visits by each modern FP method from the Uganda HMIS. The HMIS summary reports provide information on new and revisiting clients for each modern FP method (pills, injectables, condoms, emergency contraception, implants, intrauterine devices, and sterilizations). We used these data for our analysis because although HMIS summary reports also include data on contraceptive methods dispensed, in discussion with the country team, we determined there are challenges with the quality of this indicator. This is also consistent with the indicator that the national and local FP programs use for tracking their progress in contraceptive uptake.

The HMIS data the authors used were disaggregated by age (10–24 years, 25–49 years). While the focus of TCI’s AYSRH programming in the 3 local governments was specifically for adolescents and youth aged 15–24 years, Uganda’s HMIS FP data can only be disaggregated as 10–19 years, 20–24 years, and 25–49 years. It is not possible to further break down the FP data among adolescents and youth to exclude those aged 10–14 years from the analysis. Thus, the quantitative analysis focused on adolescents and youth aged 10–24 years as well as older women aged 25–49 years.

To understand the differences among the local governments with the AYSRH intervention and other local governments and Uganda, we analyzed 3 subsets of the HMIS data:

1. General FP+AYSRH intervention sites: Three local governments where AYSRH was layered on the general FP program
2. General FP only sites: Eleven local governments with the general FP program
3. Uganda country total

Before analyzing the data, the authors reviewed the data quality of the FP service statistics collected from the HMIS database—including missing data, positive or negative outliers, and number of reporting facilities—and addressed these issues in a uniform manner. Missing data were validated for whether they were actually missing (i.e., whether the data were actually available but not reported) or if zero was actually the intended entry (i.e., no service uptake for the month). Outliers were also validated and, if applicable, were updated with the correct data or accompanied by explanations.

For this article, we focused on calculating numbers for the indicator called net contraceptive uptake (NCU), defined as the derived number of additional modern FP clients over a particular period of interest, expressed in annualized terms per 100 WRA. NCU was developed by TCI to track changes in contraceptive uptake over time using service statistics data instead of relying on population-based surveys. We used this indicator instead of standard indicators like modern contraceptive prevalence rate because service statistics data were widely available. Moreover, these data are reported by facilities to the HMIS database and accessed by TCI on a monthly basis, allowing for near-real-time monitoring of the trends.

The subanalysis for the different age groups (10–24 years and 25–49 years) uses the corresponding population sizes for those age groups. NCU accounts for the duration of protection provided by short-acting methods and the rate of discontinuation exhibited by users of long-acting reversible contraceptive methods. NCU does not include data for traditional methods and focuses on modern FP methods only. More information on TCI’s contraceptive uptake indicators has been published by Finkle et al.^22^ and in Supplement 1.

### Net Contraceptive Uptake Trend Analysis

We calculated NCU for each implementation year, for each age category, and by category, namely: (1) general FP+AYSRH, (2) general FP only program, and (3) the entire country of Uganda. We then plotted the annual trends of the 3 categories across the 2 age groups to compare if changes seen since the start of TCI engagement were comparable. NCU analysis is critical in determining whether the program areas had increases in FP uptake in each of the 3 local government categories and how those changes compare with each other.

### Difference-in-Difference Analysis

A difference-in-difference (DID) analysis was then conducted to compare quantitative changes in NCU among the 3 local government categories based on when AYSRH layering began. For each age group (10–24 years and 25–49 years), we calculated the DID analysis as follows. Difference 1 is the difference between the average NCU after intervention (July 2018–June 2021) and the average NCU before intervention (July 2016–June 2018) in general FP+AYSRH local governments, general FP only local governments, and Uganda country total. The DID is the difference between difference 1 for general FP+AYSRH and general FP only and general FP only and Uganda country total. We called these the “advantage” numbers because they show whether the before-and-after difference seen in the general FP+AYSRH local governments is higher than the difference seen in general FP only local governments, as well as whether the before-and-after difference seen in the general FP only local governments is higher than the difference seen in the Uganda country total.

Given that we used service statistics data from HMIS, which records all women visiting the facilities for FP services, it was not relevant to apply statistical tests of significance for our analysis. As Charles Cowger wrote^30^:

*Significance tests are not only inappropriate when applied to a total population but are unnecessary since the probable relation of a sample and a population is defined as unity when they are the same.*

## RESULTS

### Net Contraceptive Uptake Among Adolescents and Youth Aged 10–24 years

First, we focus on answering whether layering AYSRH interventions onto an existing and functioning FP program targeting women aged 15–49 years results in an increased contraceptive uptake among youth.

**FIGURE 1:**
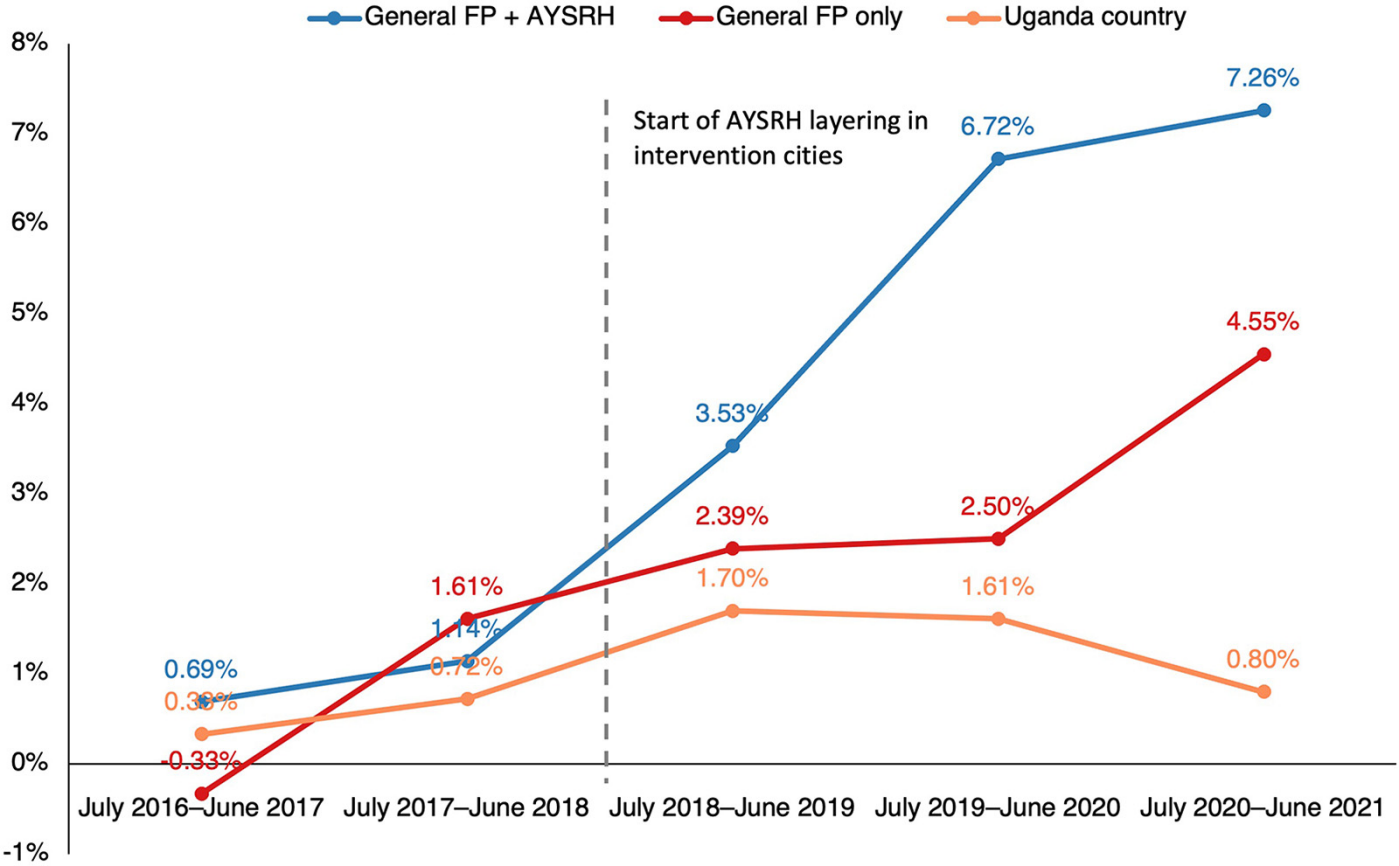
Comparison of Net Contraceptive Uptake Trends Among Adolescents and Youth Aged 10–24 Years Abbreviations: AYSRH, adolescent and youth sexual and reproductive health; FP, family planning.

Before TCI began implementation, the NCU was roughly similar in general FP+AYSRH local governments, general FP only local governments, and the country overall (Figure 1). However, after TCI began its implementation, NCU was higher in general FP only local governments compared to the country total. Moreover, after TCI-supported implementation, NCU was higher in general FP+AYSRH local governments than both general FP only local governments and the country total. This is evidence of increasing contraceptive uptake among adolescents and youth aged 10–24 years, with bigger changes seen in general FP+AYSRH local governments compared to general FP only local governments and the country overall. Interestingly, within the latest year, a more notable increase in NCU was seen in the general FP only local governments compared to previous years, while a positive NCU trend was still maintained for general FP+AYSRH local governments, albeit at a decreased slope. Supplement 1 includes the breakdown of NCU calculations.

After TCI implementation, NCU for both adolescents and youth aged 10–24 years and women aged 25–49 years was higher in general FP+AYSRH local governments than both general FP only local governments and the country total.

### Net Contraceptive Uptake Among Women Aged 25–49 Years

Second, we focus on whether layering AYSRH interventions onto an existing and functioning FP program also increases contraceptive uptake among women aged 25–49 years.

**FIGURE 2:**
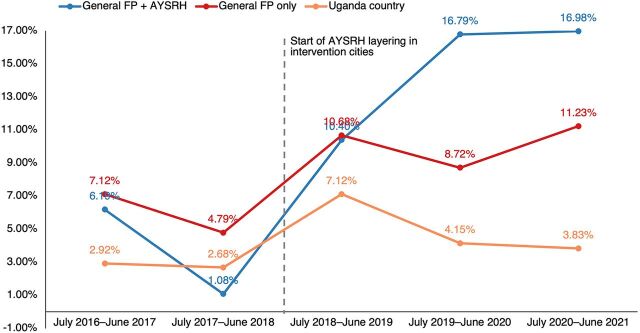
Comparison of Net Contraceptive Uptake Trends Among Women Aged 25–49 Years Abbreviations: AYSRH, adolescent and youth sexual and reproductive health; FP, family planning.

Before TCI implementation, the data show that NCU was higher in general FP only local governments compared to general FP+AYSRH local governments or the country total (Figure 2). However, this changed after TCI began implementation—with general FP+AYSRH local governments showing higher NCU than general FP only local governments and the country total. This supports a boost—notable increases in contraceptive uptake—among older women aged 25–49 years in TCI local governments where AYSRH was layered (Supplement 2 includes the breakdown of NCU calculations). Notably, within the latest year, the NCU increased for the general FP only local governments, especially after the drop observed in the previous year. Meanwhile, the notable NCU increase observed in general FP+AYSRH local governments was maintained when comparing the last 2 years.

### Difference-in-Difference Analysis

A DID analysis showed that among the 10–24 year age group, there was a 1.7 percentage point advantage of general FP only local governments over the country total and a 2.4 percentage point advantage of general FP+AYSRH local governments over general FP only local governments (Table 3). Among the women aged 25 years and older, there was a 2.0 percentage point advantage of general FP only local governments over the country total and a 6.8 percentage point NCU advantage of general FP+AYSRH local governments over general FP only local governments. These results illustrate an overall performance edge in TCI general FP+AYSRH local governments over general FP only local governments, which, in turn, outperform the rest of the country. This was consistent for both adolescents and youth (aged 10–24 years) and older women (aged 25–49 years).

**TABLE 3.** Difference-in-Difference Analysis Results Based on Intervention Implementation

	**Yearly Net Contraceptive Uptake Average**							
	**Aged 10–24 Years**				**Aged 25–49 Years**			
	**Before**	**After**	**Difference**	**Difference-in-Difference**	**Before**	**After**	**Difference**	**Difference-in-Difference**
General FP+AYSRH local governments	0.9	5.8	4.9	2.4^a^	3.6	14.7	11.1	6.8^a^
General FP only local governments	0.6	3.1	2.5	1.7^b^	6.0	10.2	4.3	2.0^b^
Uganda country total	0.5	1.4	0.8		2.8	5.0	2.2	

Abbreviations: AYSRH, adolescents and youth sexual and reproductive health; FP, family planning.

^a^ General FP+AYSRH local governments minus general FP only local governments.

^b^ General FP only local governments minus Uganda country total.

We did not calculate a statistics test of significance because we used FP service statistics data from HMIS—these are based on the actual number of new and revisiting FP clients seen in facilities. We did not use a random sample from population-based survey data.

## DISCUSSION

This article shows that layering an AYSRH program onto a well-functioning FP program not only increases contraceptive uptake for adolescents and youth but also boosts uptake for older women 25–49 years. For both age groups, the increases in contraceptive uptake were greater in general FP+AYSRH local governments compared to general FP only local governments and the country totals.

There are several factors that likely contributed to this unanticipated boost. When layering AYSRH onto the general FP program, advocacy and funding increased in the 3 local governments (Buikwe, Iganga, and Mukono) that had already used funding for the general FP programs. The additional TCI funding^23^ for AYSRH provided more support to existing systems at the governance, health system, and community levels.

At the governance level, the district leadership got the opportunity to drive the AYSRH agenda along with FP. This motivated and inspired them to commit financial as well as human resources toward reaching neglected adolescents and youth with contraceptive services that were now being delivered with quality standards. The 3 local governments that implemented AYSRH-specific services advocated for and secured additional funds for training providers; improving facility readiness for contraceptive provision to youth, including holding youth-specific whole-site orientations; conducting more demand generation activities in advance of outreach services; and hosting intergenerational dialogue with community gatekeepers. This was motivating for committing financial and human resources toward targeted efforts to reach neglected adolescents and youth, who are no doubt an important voter base and whose well-being also genuinely promises a healthier and economically more prosperous future for current and future generations.

While the additional community-level demand generation and outreach services focused on youth, there was no age cutoff for women attending, so women aged 25 years and older attended, listened, and received services. Similarly, FP-related outreach activities intended for those aged 15–24 years were also a welcome resource for those older than 24 years. While the spread of ideas between these 2 age cohorts of youth and older women has been illustrated in some recent analyses, the focus has mostly been on older women potentially influencing younger women, particularly on uptake of contraception.^31^ However, our results suggest that adding a focus on AYSRH in communities that already had a functioning FP program resulted in dual benefits with increases in contraceptive uptake for women aged 15–24 years and an unexpected boost in contraceptive use among women 25–49 years—and this increase occurred at greater rates than in areas where only a general FP program was implemented.

Community-level demand generation and outreach services focused on youth, there was no age cutoff, so women aged 25 years and older attended as well.

It is possible that TCI’s investment in ensuring community “preparedness” and developing support for somewhat controversial AYSRH in these 3 urban local governments increased supportive norms for contraceptive uptake for all women. In this case, audience segmentation to benefit AYSRH had a boost effect on older women already exposed to FP messages and services. The AYSRH services that were layered onto general FP services were most likely accessed by older women, too. However, reconstructing such project-level data retrospectively with age disaggregation was not possible for us. Communities that are more supportive of contraceptive access for youth would provide a more conducive normative culture for older women to do the same, while youth may still face residual barriers. The intergenerational dialogues that were also held in these communities with women and men attending may not have induced the most disapproving gatekeepers to attend. Future program efforts need to systematically build in well-defined qualitative research to document and analyze such factors from the perspectives of older cohorts and gatekeepers as well.

At the facility level, provider bias on FP is well documented,^32^ including age-based discrimination against adolescents and youth. TCI supported provider bias reduction through values clarification training followed by whole-site orientations, enabling health care providers and facilities to offer higher quality, nonjudgmental, and respectful AYSRH services to adolescents and youth as well as all women. Respectful and nonjudgmental care provided with assurance of confidentiality and privacy is critically important for adolescents and youth but highly valued by all age groups, and this could further improve the acceptability of outreach and facility-level FP services for older women.

Within the last year, it was observed that the NCU had a lower, though still slightly positive, slope for adolescents and youth as well as older women aged 25–49 years in the general FP+AYSRH local governments (as noted in the Results). This suggested that in the 3 FP+AYSRH local governments, there may have been some challenging systemic issues, perhaps COVID related, that adversely affected access to contraceptives for both age cohorts. It could also be possible that a sufficient proportion of unmet need may have been addressed in these 3 general FP+AYSRH local governments, lowering demand. In contrast, the more persistent increase in NCU in the 11 general FP only local governments in the same time frame suggested that they could have benefited from diffusion from the 3 general FP+AYSRH local governments, with pent up unmet need then being acted on by more informed clients and better-prepared health workers. In the future, this also should be systematically tracked for contiguous areas, in this case, to the 3 general FP+AYSRH local governments. Adolescents and youth now are certainly connected through cell phones and social media, which those aged 25–29 years may also be practicing, though perhaps not as much for those aged 30–49 years. Moreover, health workers are also community members who share good practices, and the diffusion effects may have influenced their service delivery culture to be nonjudgmental, respectful, and inclusive in the contiguous local governments.

For sustainability, the local governments need to ensure that the adolescent and youth structures within the health system are well functioning and well resourced, so that they can continue advocacy with the community gatekeepers and local leaders for a more enabling environment and for youth-responsive health services in the facilities. Also, future programming to improve contraceptive uptake should consider TCI’s evolved approach, which is now to consciously integrate a focus on youth for AYSRH as well as older women aged 25–49 years in the same communities to benefit from such boost effects. This builds a more cohesive, cost-effective, and more sustainable approach to supporting the entire health services delivery team as well as community-level demand generation and outreach efforts, complemented by intergenerational dialogue to address biases for all WRA with again a special focus on adolescents and youth.

### Limitations and Future Research

One main limitation of the study is the relatively small number of intervention local governments (3 general FP+AYSRH local governments) as well as the short time frame, so it will be beneficial to see if similar results are observed in more TCI local governments implementing the AYSRH layering and over a longer period of time. Although the use of service statistics data from HMIS provides various strengths to this study, there are also several limitations. The study focuses on HMIS variables on contraceptive uptake—specifically new and revisiting clients by each modern FP method. Summary HMIS data have limited demographic information (e.g., age) that can be further used in this study. There is also no household survey data available at the local government level to run regressions on the factors that may be affecting the results. Future research that can triangulate the data from HMIS to other surveys or additional facility-based information could be useful. In addition, there was a limitation in analyzing the uptake of traditional methods because the NCU indicator focuses only on modern contraceptive methods. Although TCI’s AYSRH programming focused on adolescents and youth aged 15–24 years, HMIS data from Uganda only allows for disaggregation for the age groups of 10–19 years and 20–24 years; thus, the quantitative analysis included this broader age group. Missing data and outliers were corrected as needed, but otherwise, the quality of HMIS data and consistency of reporting over time were some factors that could have affected the level of differences observed in the study. Moreover, the study focused on comparing against national totals, but further analysis could also look into comparisons with other urban portions of the country. Also, additional analysis of the differences in contraceptive uptake by method type (short-acting, long-acting reversible, and permanent methods) can be of interest in future studies. Finally, because the boost in contraceptive uptake among women aged 25–49 years was unanticipated, TCI Uganda project records were not set up to capture this with appropriate age disaggregation or tracking of diffusion (or “reverse diffusion”) networks. Thus, community norms and perceptions on contraception for women aged 15–24 years and the perspectives of women aged 25–49 years on “more acceptability” for their contraceptive use after the addition of the focus on AYSRH were not explored.

## CONCLUSION

This article demonstrates how layering an AYSRH program onto a well-functioning general FP program not only contributes to increased contraceptive uptake among adolescents and youth but also boosts uptake among women 25 years and older. This suggests that AYSRH programs could take advantage of the foundation of FP programs where they exist and add youth-specific interventions that typically operate through similar channels but with segmentation of the approaches.

## Supplementary Material

GHSP-D-22-00308-supplement.pdf

## Notes

**Peer Reviewed**

**First Published Online:** April 18, 2024.
