# Supplementary material for: Boosting Contraceptive Uptake in Urban Uganda: Older Women Benefit When Layering Adolescent and Youth Interventions Onto Existing Family Planning Programming
Source: Glob Health Sci Pract. 2024 May 21;12(Suppl 2):e2200308. doi: 10.9745/GHSP-D-22-00308 (PMC11111102; doi:10.9745/GHSP-D-22-00308)
Supplement: GHSP-D-22-00308-supplement.pdf [file GHSP-D-22-00308-supplement.pdf]

Supplement to: Bwire A, Sama DJ, Mirano J, et al. Boosting contraceptive uptake in urban Uganda: older women benefit when layering adolescent and youth interventions onto existing family planning programming. *Glob Health Sci Pract*. 2024;12(Suppl 2):e2200308. https://doi.org/10.9745/GHSP-D-22-00308

Supplement 1. Net Contraceptive Uptake Description and Processing

TCI extracted the number of FP service visits data from the HMIS database in Uganda. This was collected by individual contraceptive methods (e.g. condoms, pills, emergency contraception, injectables, IUD, implants, sterilization) and aggregated into three method types: short-acting methods (condoms, pills, emergency contraception, injectables), long-acting reversible contraceptives or LARCs (IUD, implants), and permanent methods (sterilization). Prior to processing the raw data, TCI reviewed the data quality of FP service statistics collected from the HMIS database – including missing data, positive or negative outliers, and number of reporting facilities – and addressed these issues in a uniform manner across different countries.

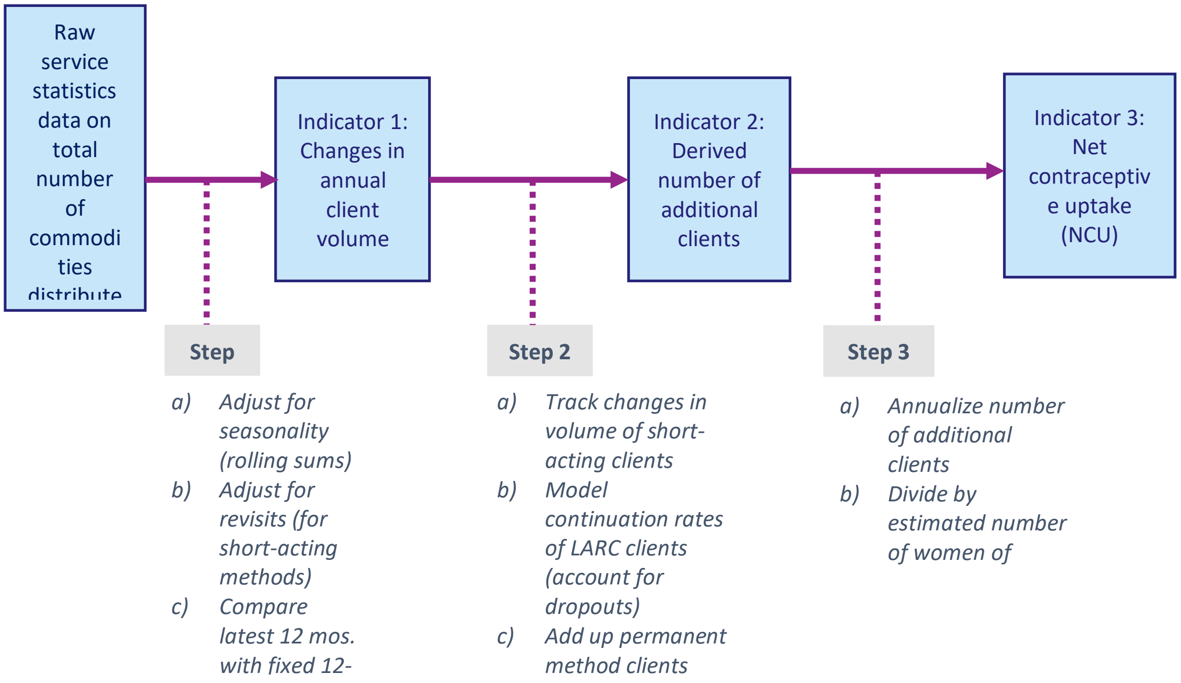

Figure 1 TCI's methodology of analyzing service statistics data

The approach we used filters the noise in service statistics data coming from seasonality by using 12-month rolling sums. CYP adjustment factors were also applied to the number of short-acting methods availed, but not for LARCs or permanent methods. This is because at this point, we are interested in estimating the number of clients accessing a family planning method in a given 12-month period; the number of short-acting methods availed were discounted according to the expected refill patterns or repeat visits within a year, while LARC or permanent method clients were counted only once since they are not expected to return within the same year (adjustments for discontinuation of LARCs were applied to the next indicator). The resulting indicator, **client volume,** is a programmatic indicator of the annual inflow of clients into the pool of contraceptive

users. It is defined as *the equivalent number of women who received family planning methods in the past twelve months.* This indicator is a measure of 12-month incidence, not prevalence, and TCI views it as more meaningful than CYP itself because it does not unduly inflate the contribution of LARCs and permanent methods in the year they were availed. A useful extension to this indicator is expressing client volume in relation to the number of women of reproductive age (15-49) in the area of interest. This standardization is helpful when comparing data across different areas.

Box 1: Definitions of TCI's service statistics-based indicators

Annual family planning client volume: the number of women who received a family planning method in the past twelve months, with those receiving short-acting methods expressed in CYP equivalents

Derived number of additional family planning clients since month A up to month B: the number of women who previously received a family planning method and assumed to be using it at month B, less the number of women who previously received a family planning method and assumed to be using it at month A-1, with assumptions on use based on method type

Net contraceptive uptake (NCU) since month A up to month B: for the relevant period, the annualized number of additional family planning clients per 100 women of reproductive age (15-49 years old)

The next step was to integrate into the analysis the outflow of clients from the pool of contraceptive users by considering the refill needs of previous clients using short-acting methods and typical discontinuation patterns of previous clients who have adopted LARCs. By doing this calculation for the latest month relative to a baseline month (before a city's engagement with TCI), one can estimate the **derived number of additional clients** over that period of interest. This indicator is derived from service statistics data on the inflow of clients into the pool of contraceptive users, but now coupled with assumptions on the outflow of those same clients from the pool of contraceptive users based on the type of method they received. It should not be mistaken for additional users, which is derived from surveys by calculating the growth in the mCPR between survey rounds and using that to estimate the increment in the number of FP users over that period.

Finally, using the derived number of additional clients since TCI engagement from the previous step, we annualize these numbers and then relate them to the population of women of reproductive age (15-49) in the area of interest. This step produces the **net contraceptive uptake (NCU)**, which is the derived number of additional clients over a particular period of interest, expressed in annualized terms per 100 women of reproductive age. This indicator should not be mistaken for changes in mCPR; it is a measure of changes in incidence adjusted for discontinuation, not changes in prevalence.

The methodology applied in this study assume the following: (1) there are no changes in contraceptive uptake within the private sector (however, given complete data from the private sector, the same methodology can be applied); (2) it is acceptable to use the same LARC continuation rates in all countries; (3) there is minimal ageing out of permanent method users; and (4) there is minimal distortion in using the CYP for the most common type of injectables or implants in each country. These assumptions were informed by the near real-time program monitoring approach used by TCI, but they can be modified in future studies depending on specific objectives.

Supplement to: Bwire A, Sama DJ, Mirano J, et al. Boosting contraceptive uptake in urban Uganda: older women benefit when layering adolescent and youth interventions onto existing family planning programming. *Glob Health Sci Pract*. 2024;12(Suppl 2):e2200308. https://doi.org/10.9745/GHSP-D-22-00308

Lastly, it is important to understand the differences and complementarity of the indicators used in this study. Analyzing changes in FP client volume can be very helpful for facility and city-level goal setting since this indicator is closest to the data collected at registers and can be easily traced back to the raw data. Data on the derived number of additional clients builds on client volume by incorporating assumptions on client outflows. It provides a useful basis for monitoring progress that can be compared against intuitive benchmarks, like FP2020's goal of supporting 120 million additional users (relative to 2012 baseline) by the end of 2020.[7] With NCU, we have applied all adjustments related to seasonality, revisits, and continuation rates and have scaled it by population, making it a more meaningful summary of changes in contraceptive uptake in each city.

Supplement to: Bwire A, Sama DJ, Mirano J, et al. Boosting contraceptive uptake in urban Uganda: older women benefit when layering adolescent and youth interventions onto existing family planning programming. *Glob Health Sci Pract*. 2024;12(Suppl 2):e2200308. https://doi.org/10.9745/GHSP-D-22-00308

Supplement 2. Net Contraceptive Uptake Calculations by Age Group, Year, and City Type

AGE GROUP: 10-24 years

		July 2016-June 2017	July 2017-June 2018	July 2018-June 2019	July 2019-June 2020	July 2020-June 2021
General FP + AY (3 cities)	N	2324	3824	11860	22577	24399
	D	336036	336036	336036	336036	336036
	P	0.69	1.14	3.53	6.72	7.26
General FP only (11 cities)	N	-2815	13565	20206	21097	38379
	D	844220	844220	844220	844220	844220
	P	-0.33	0.72	1.70	1.61	0.80
Uganda Country	N	25205	54993	129845	122971	61104
	D	7637943	7637943	7637943	7637943	7637943
	P	0.33	0.72	1.70	1.61	0.80

AGE GROUP: 25-49 years

		July 2016-June 2017	July 2017-June 2018	July 2018-June 2019	July 2019-June 2020	July 2020-June 2021
General FP + AY (3 cities)	N	7834	1362	13163	21253	21495
	D	126601	126601	126601	126601	126601
	P	6.19	1.08	10.40	16.79	16.98
General FP only (11 cities)	N	22641	15230	33966	27751	35725
	D	318060	318060	318060	318060	318060
	P	7.12	4.79	10.68	8.72	11.23
Uganda Country	N	223028	204697	543822	316975	292533
	D	2877593	2877593	2877593	2877593	2877593
	P	2.92	2.68	7.12	4.15	3.83

Legend:
N – numerator – additional family planning clients within the year.
D – denominator – estimated population of women within the age group (2019).
P - proportion – net contraceptive uptake, additional clients per 100 women within the age group.
